# Head and neck cancer and occupational exposure to leather dust: results from the ICARE study, a French case-control study

**DOI:** 10.1186/s12940-019-0469-3

**Published:** 2019-03-29

**Authors:** Loredana Radoï, Fatoumata Sylla, Mireille Matrat, Christine Barul, Gwenn Menvielle, Patricia Delafosse, Isabelle Stücker, Danièle Luce, ICARE study group, Anne-Valérie Guizard, Arlette Danzon, Anne-Sophie Woronoff, Michel Velten, Antoine Buemi, Émilie Marrer, Brigitte Trétarre, Marc Colonna, Patricia Delafosse, Paolo Bercelli, Florence Molinié, Simona Bara, Bénédicte Lapotre-Ledoux, Nicole Raverdy, Sylvie Cénée, Oumar Gaye, Florence Guida, Farida Lamkarkach, Loredana Radoï, Marie Sanchez, Isabelle Stücker, Matthieu Carton, Diane Cyr, Annie Schmaus, Joëlle Févotte, Corinne Pilorget, Gwenn Menvielle, Danièle Luce

**Affiliations:** 1 Paris Sud, Paris Saclay University, UVSQ, CESP, INSERM U1018, Environmental Epidemiology of Cancer Team, Villejuif, France; 2 Oral medicine and oral surgery department, University Paris Descartes, Faculty of Dental surgery, Paris, France; 3 University Pierre et Marie Curie, Paris, France; 4 Faculty of Medicine, University Paris Est Créteil, Créteil, France; 5 Centre Hospitalier Intercommunal, Service des Pathologies Professionnelles et de l’Environnement, Créteil, France; 6 Université de Rennes, Inserm, EHESP, Irset (Institut de Recherche en Santé, Environnement et Travail)-UMR_S 1085, Pointe-à-Pitre, France; 7 Department of social epidemiology, INSERM, Sorbonne Université, Institut Pierre Louis d’Epidémiologie et de Santé Publique IPLESP, Paris, France; 8 Isère Cancer Registry, CHU Grenoble-Alpes, Grenoble; FRANCIM Network, Toulouse, France

**Keywords:** Leather dust, Occupational health, Head and neck cancer, Epidemiology, Case control study

## Abstract

**Background:**

Leather dust is an established carcinogen of the sinonasal cavities; however, evidence is lacking regarding its association with other head and neck cancers (HNC). To date, few studies have been conducted on the association between occupational leather dust exposure and the risk of oral, pharyngeal, and laryngeal cancers. The objective of this study was to investigate the association between the risk of HNC and occupational exposure to leather dust.

**Methods:**

Lifestyle habits and occupational history were collected for 2161 patients with squamous cell carcinoma of oral cavity, pharynx, and larynx, and 3555 controls, using a standardized questionnaire. Occupational exposure to leather dust was assessed using a job-exposure matrix. Odds ratios (OR) and 95% confidence intervals (CI) for HNC globally and by subsite were estimated using multivariate unconditional, and polytomous logistic regressions, respectively.

**Results:**

Cumulative lifetime exposure to leather dust < 6 mg/m^3^-years was associated with an increased risk of laryngeal cancer (OR = 2.26, 95% CI: 1.07–4.76); higher levels were not related to elevated risks of HNC. Some tasks performed and the use of some glues were associated with elevated, although non-significant, risks of HNC. No dose-response relationships were observed.

**Conclusion:**

Our study did not provide enough evidence for an increased risk of HNC related to occupational exposure to leather dust. Further studies are needed to understand the risks of specific tasks in the leather industry.

**Electronic supplementary material:**

The online version of this article (10.1186/s12940-019-0469-3) contains supplementary material, which is available to authorized users.

## Introduction

Head and neck cancers (HNC; excluding sinonasal cancers) accounted for an estimated 11,240 new cases of cancer in France in 2012; this is the third-highest incidence rate and the fourth most frequent cancer among men in Europe [1]. Tobacco smoking and alcohol consumption are well-known major risk factors for oral cavity, pharyngeal and laryngeal cancers [2, 3]. There is also evidence that other factors may contribute to these diseases, including oropharyngeal infection with human papillomavirus (HPV) [4, 5], unbalanced diet [6], poor oral health [7], and low socioeconomic position [8–10]. Exposures to asbestos and strong acid mists are known to be occupational risk factors for laryngeal cancer [11, 12]. Furthermore, risk of laryngeal cancer was associated with exposure to polycyclic aromatic hydrocarbons, engine exhaust, textile dust, and working in the rubber industry [13]. Exposures to asbestos and polycyclic aromatic hydrocarbons were found to be related to an increased risk of oral and pharyngeal cancers [14].

The International Agency for Research on Cancer (IARC) classified occupational exposure to leather dust as carcinogenic to humans [15]. Indeed, there is consistent and strong evidence from epidemiological studies that exposure to leather dust causes cancer of the nasal cavity and paranasal sinuses, particularly in workers employed in the boot and shoe industry [15].

However, studies on occupational exposure to leather dust causing other HNC are conflicting. Only two studies reported results for employment in the leather and leather product industries alone: a case control-study observed slightly more cases of cancers of the oral cavity, pharynx and larynx than expected [16]; a cohort study reported no increased risk of laryngeal cancer [17]. Other studies have explored associations between the risk of HNC and exposure to leather dust, or occupations related to leather work; a case-control study observed a significant excess risk of pharyngeal squamous cell carcinoma associated with exposure to leather dust [18]; two case-control studies reported significantly higher risks of laryngeal and hypopharyngeal cancer for shoe finishers, but not for shoe makers or repairers [19], or for leather workers in general [20]. In addition, a cohort study reported a significantly elevated risk of oral and pharyngeal cancers among shoemakers/cobblers, but no association with exposure to leather dust [21]. Conversely, several studies did not find increased risks of HNC associated with exposure to leather dust, or occupations related to the leather work [17, 22–25].

Using the data from the ICARE study (**I**nvestigation of occupational and environmental **CA**uses of **RE**spiratory cancers study), we aimed to: (i) examine the association between the risk of HNC and occupational exposure to leather dust, overall and by subsite; (ii) analyze this association through materials used, tasks performed, and co-exposure to glues.

## Methods

### ICARE study population

The design of the study has already been reported [26]. Briefly, the ICARE study is a multicenter, population-based case-control study, conducted between 2001 and 2007, comprising cases of HNC (*n* = 2415) and lung cancer (*n* = 2926), and controls (*n* = 3555). Incident cases were identified in collaboration with the French cancer registries in 10 geographical areas. They were all aged 18–75 years at diagnosis, with histologically confirmed primary tumors. Controls were selected from the general population of the geographical areas included in the study by list-assisted random digit dialing sampling and an incidence density sampling method. They were frequency-matched to all cases (lung cancer and HNC) by sex and age. Additional stratification was used to achieve a socioeconomic status (SES) distribution among the controls comparable to that of the general population.

### Data collection

Using a standardized questionnaire, subjects were interviewed face-to-face by trained interviewers to collect information on sociodemographic characteristics, anthropometric measurements, personal and familial history of cancer, lifetime tobacco and alcohol consumption, and entire occupational history. An occupational history questionnaire was designed with the collaboration of industrial hygienists. It included general questions on occupational history. For each job held for at least 1 month, the subject was asked for the job title, duration of employment, activity sector, main and subsidiary tasks, and the description of the work environment. Specific questionnaires (covering 20 job titles, including work in the leather industry) were also designed with more technical questioning.

A shorter version of the general questionnaire was used for subjects who were too sick to answer the complete questionnaire (10.8% of cases, and 2.1% of controls). This shorter questionnaire (answered by the subjects themselves or by their next-of-kin) included mainly information on tobacco and alcohol consumption, and occupational history, without detailed questions on each job held or tasks performed. Therefore, for these subjects, occupations and industries were recovered from the analyses of the general questionnaire. However, they were excluded from the analysis of the leather work questionnaire due to a lack of response to this questionnaire.

Participation rates were 80.6% among controls, and 82.5% among cases. Each subject gave written informed consent. The study was approved by the Institutional Review Board of the French National Institute of Health and Medical Research (IRB-Inserm, no. 01–036), and by the French Data Protection Authority (CNIL no. 90120).

### Study sample

The present analysis was restricted to the following HNC sites as coded by the International Classification of Diseases for Oncology (ICD-O-3) [27]: oral cavity (C00.3-C00.9, C02.0-C02.3, C03.0, C03.1, C03.9, C04.1, C04.8, C04.9, C05.0, C06.0-C06.2, C06.8 and C06.9); oropharynx (C01.9, C02.4, C05.1, C05.2, C09.0, C09.1, C09.8, C09.9, C10.0-C10.4, C10.8, C10.9); hypopharynx (C12.9, C13.0-C13.2, C13.8, C13.9); oral cavity/pharynx unspecified or overlapping (C02.8, C02.9, C05.8, C05.9, C14.0, C14.2, C14.8), and larynx (C32.0-C32.3, C32.8, C32.9). Among the 2237 cases of HNC, 2161 (96.6%) were squamous cell carcinomas and only 76 (3.4%) presented other histological types (mainly unspecified carcinomas, adenocarcinomas, and other specified carcinomas). Of the 76 cases with other histological types, only one was exposed to leather dust; therefore, analysis was restricted to squamous cell carcinomas (*n* = 2161) and all controls (*n* = 3555).

### Coding of job titles

All jobs held for at least 1 month during the working life of the subjects were coded according to the International Standard Classification of Occupations (ISCO, 1968) [28] for occupations, and to the French Nomenclature of Activities (NAF, 2008) [29] for industries.

### Assessment of occupational exposure to leather dust

Occupational exposure to leather dust was first assessed using a job-exposure matrix (JEM) for inhalable leather dust (particles < 100 μm) [30]. For each combination of ISCO and NAF codes, the JEM provided two indices: the probability of exposure (the percentage of exposed workers), and level of exposure. Then, using lifetime occupational history and indices of exposure provided by the JEM, the following exposure variables were computed: ever exposure to leather dust (“ever” defined as having worked in at least one job with a non-zero probability of exposure), maximum probability of exposure, maximum level of exposure, cumulative duration of exposure, and cumulative exposure index (CEI). The maximum probability and level of exposure were the highest probability and level measured over the entire occupational history of each subject. The cumulative duration of exposure was calculated by summing the periods in which the subject was exposed. CEI was estimated by the sum of the multiplication of the weighted duration, probability, and level of exposure on the entire professional life (see Additional file 1: Table S1).

For each job period involving leather work (shoemaking, and manufacture of leather clothes and other leather goods), a specific questionnaire was administered only to subjects that filled out a complete general questionnaire; it included questions about the materials handled, tasks performed, and exposure to several types of glues. Information from this specific questionnaire was analyzed in addition to the exposure assessment with the JEM.

### Statistical analysis

Unconditional multivariable logistic regression was used to estimate odds ratios (ORs) and corresponding 95% confidence intervals (95% CI) of HNC associated with occupational exposure to leather dust. ORs were also estimated for each cancer subsite using multinomial (polytomous) logistic regression. All ORs were adjusted for age (< 51, 51–58, 59–65, and ≥ 66 years), sex, area of residence (10 geographical areas), SES (assessed by the longest held occupation), tobacco smoking status (never; former: cessation ≥ 2 years at the interview for controls, or at diagnosis for cases; current), tobacco consumption in pack-years (never, < 7, 7–16, 17–29, > 29), and alcohol drinking in glass-years (never, < 9, 9–46, 47–118, > 118). Categories for tobacco and alcohol consumption were based on the distribution of exposed controls, with quartiles used as cutoff points. Maximum probability and maximum level of exposure to leather dust were categorized as following: 0 (not exposed), 1–50, > 50%; and 0 (not exposed), 1–2.9 mg/m^3^, ≥3 mg/m^3^, respectively. Categories for the duration of exposure to leather dust and the CEI were based on the distribution of exposed controls, with medians used as cutoff points. Dose-response relationships between each variable of exposure to leather dust and HNC risk were explored using tests for linear trends by modelling the median of each category as a continuous variable. Analyses were also stratified on sex, and alcohol and tobacco status. Interactions between exposure to leather dust and sex, tobacco smoking, or alcohol drinking were tested using the test of maximum likelihood.

In addition, we performed analyses taking into account a lag time of 10 years by excluding exposure occurring 10 years before the diagnosis of cancer for cases, or the date of the interview for controls. Analyses by age at first exposure, time since first exposure and time since last exposure were also performed.

All tests were two-sided and a *p* value ≤0.05 was the threshold for statistical significance. Analyses were performed using the STATA software, version 12.1 (StataCorp, Texas, USA).

## Results

The main characteristics of the subjects included in the analysis are presented in Table 1. Cases were slightly younger than controls (average age: 57.8 vs. 58.5 years). Men represented more than two thirds of both cases and controls. The control group generally had a higher SES compared to cases (*p* <  0.0001). As expected, cases were more frequently current smokers and consumed more tobacco and alcohol than controls (*p* <  0.0001). Cancers of the oral cavity, oropharynx, hypopharynx, oral cavity/pharynx unspecified, and larynx represented 20.5, 30.5, 18.7, 6.8, and 23.5%, respectively, of all squamous cell carcinomas included in the present study.

**Table 1:** Main characteristics of subjects included in the analysis and cancer subsites

	Cases (*n* = 2161)		Controls (*n* = 3555)		*p*-value of chi 2 test
	n	%	n	%	
Age at interview (years, quartiles)					0.003
Mean (SD)	57.8 (8.6)		58.5 (10.2)		
< 51	467	21.6	899	25.3	
51–60	767	35.5	812	22.8	
59–65	508	23.5	801	22.5	
≥66	419	19.4	1043	29.3	
Sex					< 0.0001
Male	1862	86.2	2780	78.2	
Female	299	13.8	775	21.8	
Area of residence					< 0.0001
Bas-Rhin	232	10.7	469	13.2	
Calvados	210	9.7	462	13.0	
Doubs, Territoire de Belfort	47	2.2	143	4.0	
Hérault	227	10.5	450	12.7	
Isère	250	11.6	501	14.1	
Loire Atlantique	359	16.6	404	11.4	
Manche	238	11.0	312	8.8	
Haut-Rhin	39	1.8	118	3.3	
Somme	394	18.3	499	14.0	
Vendée	165	7.6	197	5.5	
Socioeconomic status (the longest held job)					< 0.0001
Managers	134	6.2	618	17.4	
Farmers	51	2.4	197	5.5	
Self-employed workers	136	6.3	177	4.9	
Intermediate white-collar workers	218	10.1	695	19.5	
Office and sales employees	341	15.8	672	18.9	
Blue-collar workers	1241	57.4	1178	33.1	
Tobacco smoking status					< 0.0001
Never smokers	110	5.1	1262	35.5	
Former smokers ^a^	589	27.3	1461	40.1	
Current smokers	1452	67.2	820	23.1	
Tobacco-smoking (pack-years, quartiles)					< 0.0001
Mean (SD)	38.3 (25.2)		13.2 (18.4)		
Never smokers	110	5.1	1262	35.5	
< 7	94	4.3	581	16.6	
7–16	174	8.1	566	16.1	
17–29	376	17.4	535	15.2	
> 29	1361	62.9	566	16.1	
Alcohol drinking (glass-years, quartiles)					< 0.0001
Mean (SD)	213.1 (233.6)		78.3 (120.1)		
Never drinkers	103	4.8	306	8.6	
< 9	149	6.9	803	22.6	
9–46	174	8.1	806	22.7	
47–118	378	17.5	804	22.6	
> 118	1273	58.9	802	22.6	
Cancer subsites^b^					
Oral cavity	444	20.5	–	–	
Oropharynx	658	30.5	–	–	
Hypopharynx	405	18.7	–	–	
Oral cavity/pharynx unspecified	148	6.8	–	–	
Larynx	506	23.5	–	–	

^a^ Former smokers were subjects who had stopped smoking at least two years before the interview (controls) or diagnosis (cases)

^b^ Only squamous cell carcinomas were included in the study

Fifty-seven cases (2.6%) and 75 controls (2.2%) held at least one job entailing exposure to leather dust (Table 2). Exposure status, maximum probability of exposure, and maximal level of exposure to leather dust did not differ significantly between cases and controls (*p* = 0.21, 0.38, and 0.16, respectively). Nevertheless, the mean of cumulative duration of exposure among exposed cases was significantly lower than that of exposed controls (5.9 vs. 9.9 years; *p* = 0.008). In addition, the mean of CEI was not significantly lower among exposed cases than that of exposed controls (12.3 mg/m^3^-years vs. 27.2 mg/m^3^-years; *p* = 0.06).

**Table 2:** Association between the risk of head and neck cancer, globally and by subsite, and exposure to leather dust assessed using a job-exposure matrix. Results from the general questionnaire

	Controls	HNC overall		Oral cavity		Oropharynx		Hypopharynx		Oral cavity, pharynx unspecified		Larynx	
	n	n	OR^a^ (95% CI)	n	OR^a^ (95% CI)	n	OR^a^ (95% CI)	n	OR^a^ (95% CI)	n	OR^a^ (95% CI)	n	OR^a^ (95% CI)
Exposure to leather dust													
Never exposed ^b^	3476	2087	reference	433	reference	643	reference	386	reference	141	reference	484	reference
Ever exposed ^c^	75	57	0.99 (0.62, 1.56)	9	0.71 (0.32, 1.58)	12	0.64 (0.31, 1.29)	12	1.24 (0.61, 2.52)	5	1.47 (0.54, 3.99)	19	1.40 (0.77, 2.56)
Maximum probability of exposure (%)													
≤ 50	32	26	1.17 (0.59, 2.31)	3	0.46 (0.10, 2.08)	8	1.21 (0.49, 2.94)	3	0.85 (0.23, 3.07)	3	2.36 (0.63, 8.78)	9	1.60 (0.66, 3.85)
> 50	43	31	0.86 (0.47, 1.58)	6	0.86 (0.33, 2.22)	4	0.28 (0.08, 0.97)	9	1.44 (0.61, 3.41)	2	0.93 (0.20, 4.22)	10	1.26 (0.56, 2.83)
Maximum level of exposure (mg/m^3^)													
< 3	50	41	1.09 (0.63, 1.89)	5	0.55 (0.18, 1.62)	10	0.82 (0.37, 1.81)	8	1.33 (0.56, 3.14)	4	1.90 (0.62, 5.87)	14	1.58 (0.77, 3.24)
≥ 3	25	16	0.79 (0.35, 1.77)	4	1.01 (0.31, 3.30)	2	0.32 (0.07, 1.48)	4	1.09 (0.32, 3.62)	1	0.75 (0.09, 6.25)	5	1.07 (0.36, 3.17)
Cumulative duration of exposure (years)													
≤ 7	43	45	1.22 (0.70, 2.14)	6	0.75 (0.27, 2.04)	8	0.64 (0.26, 1.56)	11	1.75 (0.79–3.86)	4	1.95 (0.63, 6.02)	16	1.86 (0.92, 3.75)
> 7	32	12	0.63 (0.28, 1.44)	3	0.67 (0.18, 2.46)	4	0.69 (0.22, 2.16)	1	0.34 (0.04, 2.71)	1	0.75 (0.08, 6.42)	3	0.68 (0.19, 2.45)
Cumulative exposure index (mg/m^3^-years)													
≤ 6	38	37	1.43 (0.78, 2.61)	5	0.81 (0.26, 2.47)	8	0.81 (0.33, 1.98)	6	1.42 (0.53, 3.78)	4	3.04 (0.97, 9.54)	14	2.26 (1.07, 4.76)
> 6	37	20	0.60 (0.29, 1.22)	4	0.58 (0.18, 1.82)	4	0.37 (0.12, 1.15)	6	1.01 (0.36, 2.77)	1	0.40 (0.05, 3.25)	5	0.65 (0.23, 1.85)

^a^ ORs (odds ratios) adjusted for age, sex, area of residence, socioeconomic status, and tobacco and alcohol consumption

^b^ Never exposed = reference category for all variables presented in Table 2

^c^ A subject was considered “ever” exposed to leather dust if he/she had a non-zero probability of exposure

*HNC* head and neck cancer

“Ever” exposure to leather dust was not associated with the risk of HNC, overall and by subsite (Table 2). No significant associations were found regarding the maximum probability of exposure, the maximum level of exposure and the cumulative duration of exposure, either for the HNC globally or for its subsites. Generally, the ORs were higher for the lower category of exposure. Concerning the CEI, borderline significant and significant associations were found for oral cavity/pharynx unspecified (OR = 3.04, 95% CI 0.97–9.54; *p* = 0.09) and larynx (OR = 2.26, 95% CI 1.07–4.76), but only in the lowest category of exposure. No dose-response patterns were observed.

Additional analyses conducted in those most exposed to leather dust (e.g., maximum probability > 90%, maximum level > 7 mg/m^3^, cumulative duration > 75th percentile of controls, and CEI > 90th percentile of controls) showed no associations with HNC (OR = 0.73, 95% CI 0.37–1.41; 1.36, 95% CI 0.54–3.43; 0.29, 95% CI 0.02–5.74; and 0.87, 95% CI 0.05–13.55, respectively). When all parameters were combined to define a category of subjects presenting the highest exposure profile, there were not enough subjects to conduct further analyses (0 cases and 2 controls).

Only 2 cases and 5 controls had jobs entailing exposure to leather dust, which ended less than 10 years before the diagnosis or interview; the results changed only marginally when taking into account a 10-year lag period (for example, for “ever” exposure to leather dust OR = 1.05, 95% CI 0.67–1.56 for head and neck overall, and 1.47, 95% CI 0.78–2.76 for larynx). No associations were observed regarding time since first exposure, time since last exposure, and age at first exposure (OR = 0.99, 95% CI 0.98–1.01; 0.96, 95% CI 0.75–1.22; and 1.0, 95% CI = 0.95–1.04, respectively).

The analysis stratified by sex revealed that 2.2% of men and 2.9% of women were exposed to leather dust. Even if the ORs were slightly higher among men than among women, no significant differences were observed (see Additional file 1: Table S2). The analysis stratified by tobacco and alcohol consumption revealed that 2.4% of ever smokers, 2.2% of never smokers, 2.3% of ever drinkers, and 2.2% of never drinkers were exposed to leather dust. Although ORs were slightly higher for smokers and drinkers compared to non-consumers, no significant associations were observed (see Additional file 1: Table S3 and Table S4). Interactions between gender, tobacco smoking, alcohol consumption and occupational exposure to leather dust were not significant.

Regarding the analyses performed on the data from the questionnaire on leather work, 24 cases (1.2%) and 31 controls (0.9%) filled out at least one specific questionnaire (Table 3). Compared to subjects that did not answer the questionnaire, there was no increased risk of HNC in subjects that did answer the questionnaire (OR = 1.15, 95% CI 0.57–2.31).

**Table 3:** Associations between the risk of head and neck cancer and materials handled, tasks performed, and glues used. Results from the specific questionnaire on leather work

	Cases (*n* = 1926)		Controls (*n* = 3481)		OR^a^ (95% CI)
	n	%	n	%	
Held at least one job exposed to leather dust and filled out at least one specific questionnaire					
No ^b^	1902	98.8	3450	99.1	reference
Yes	24	1.2	31	0.9	1.15 (0.57, 2.31)
Materials handled					
Exposure to soft leather					
Never	2	0.1	5	0.1	0.95 (0.16, 5.63)
Ever	19	0.9	24	0.6	1.33 (0.61, 2.92)
Exposure to thick leather					
Never	7	0.3	9	0.2	1.86 (0.55, 6.25)
Ever	14	0.7	21	0.6	0.86 (0.36, 2.06)
Exposure to crepe rubber					
Never	9	0.4	21	0.6	0.77 (0.28, 2.05)
Ever	10	0.5	7	0.2	1.93 (0.57, 6.54)
Exposure to synthetic leather					
Never	12	0.6	14	0.4	1.14 (0.41, 3.14)
Ever	10	0.5	15	0.4	1.44 (0.53, 3.86)
Exposure to textile materials					
Never	17	0.8	18	0.5	1.69 (0.70, 4.03)
Ever	6	0.3	11	0.3	0.75 (0.21, 2.56)
Exposure to other materials ^c^					
Never	15	0.7	25	0.7	0.88 (0.39, 2.01)
Ever	7	0.3	1	< 0.1	18.75 (1.77, 197.89)
Tasks performed					
Cutting of leather					
Never	5	0.2	9	0.2	1.08 (0.25, 4.51)
Ever	18	0.9	21	0.6	1.17 (0.52, 2.63)
Cutting of other materials					
Never	10	0.5	14	0.4	1.61 (0.53, 4.88)
Ever	12	0.6	16	0.4	1.03 (0.41, 2.57)
Scouring, roughing, grinding, trimming, buffing of leather					
Never	9	0.4	15	0.4	1.14 (0.39, 3.30)
Ever	13	0.6	14	0.4	1.49 (0.56, 3.92)
Gluing					
Never	6	0.3	8	0.2	2.88 (0.74, 11.2)
Ever	18	0.9	22	0.6	1.02 (0.47, 2.23)
Sewing					
Never	7	0.3	8	0.2	0.80 (0.22, 2.85)
Ever	17	0.8	23	0.6	1.34 (0.59, 3.05)
Dyeing					
Never	14	0.7	24	0.6	0.81 (0.34, 1.91)
Ever	9	0.4	6	0.1	3.22 (0.77, 13.32)
Injection of plastic materials					
Never	19	0.9	26	0.7	1.39 (0.64, 3.02)
Ever	3	0.1	3	< 0.1	0.84 (0.12, 5.95)
Other tasks ^d^					
Never	19		24		1.17 (0.54, 2.51)
Ever	2	0.1	2	0.1	8.08 (0.66, 98.21)
Glues used					
Neoprene glues					
Never	6	0.3	7	0.2	0.55 (0.14, 2.05)
Ever	18	0.9	24	0.6	1.52 (0.68, 3.39)
Solvent-based / rubber-based adhesives					
Never	6	0.3	7	0.2	1.03 (0.27, 3.92)
Ever	18	0.9	24	0.6	1.20 (0.53, 2.71)
Strong glues/animal skin glues					
Never	6	0.3	10	0.2	0.54 (0.15, 1.94)
Ever	18	0.9	21	0.6	1.60 (0.70, 3.65)
Polyurethane glues					
Never	10	0.5	7	0.2	1.87 (0.54, 6.49)
Ever	14	0.7	24	0.6	0.92 (0.39, 2.16)
Other glues					
Never	6	0.3	9	0.2	0.99 (0.28, 3.43)
Ever	17	0.8	22	0.6	1.21 (0.52, 2.82)

^a^ ORs (odds ratios) adjusted for age, sex, area of residence, socioeconomic status, and tobacco and alcohol consumption

^b^ Reference category for all analyses on materials handled, tasks performed, and glues used was the category of subjects that never held a job entailing leather dust exposure and that did not filled out (a) specific leather questionnaire(s)

^c^ Wood, cork, cardboard, twine, pitch

^d^ Shoes varnishing, polishing and brushing

Slightly elevated (although non-significant) risks of HNC were observed for exposure to soft leather (OR = 1.33, 95% CI 0.61–2.92), crepe rubber (OR = 1.93, 95% CI 0.57–6.54), and synthetic leather (OR = 1.44, 95% CI 0.53–3.86). Exposure to other materials (i.e. wood, cork, cardboard, twine, or pitch) was associated with a significantly elevated risk of HNC (OR = 18.75, 95% CI 1.77–197.89), although this was based on only 8 exposed subjects (7 cases and 1 control).

Regarding the risk of HNC associated with the tasks performed, non-significantly increased risks were observed for scouring, roughing, grinding, trimming, and buffing of leather (OR = 1.49, 95% CI 0.56–3.92), dyeing (OR = 3.22, 95% CI 0.77–13.32), and other tasks (shoes varnishing, polishing, or brushing) (OR = 8.08, 95% CI 0.66–98.21).

Exposure to neoprene glues, and strong or animal skin glues was associated with non-significantly elevated risks of HNC (OR = 1.52, 95% CI 0.68–3.39, and OR = 1.60, 95% CI 0.70–3.65, respectively).

## Discussion

The present study explored occupational exposure to leather dust in relation to HNC through the ICARE study, a large population-based case-control study in France. Exposure was assessed using a JEM and a specific questionnaire, making it possible to analyze the type of materials, tasks performed, and the concomitant exposure to several glues. Significant or borderline associations were found in lower exposure categories of CEI for the subsites: larynx, and oral cavity/pharynx unspecified. Exposure to some materials (soft leather, crepe rubber, and synthetic leather), tasks (scouring, roughing, grinding, trimming and buffing of leather, and dyeing), and glues (neoprene glues, and strong or animal skin glues) was associated with elevated, although non-significant, risks of HNC. No dose-response relationships were observed, neither in the JEM analyses nor in the specific questionnaire analyses. The majority of subjects had short-term and distant exposure to leather dust (generally, they were young apprentices, performing multiple tasks). We were unable to analyze associations for exclusive exposure to materials or glues, or exclusive tasks, because the majority of subjects had multiple occupational exposures and tasks throughout their employment.

While IARC considers leather dust to be causally related to sinonasal cancers, the literature remains sparse and inconsistent for other HNC sites. Two cohort studies in shoe-manufacturing workers [31, 32] observed slightly more cases of death by oral and pharyngeal cancer than expected, contrary to two others that observed either slightly fewer cases than expected [33] or no excess risk [34]. A census-based study observed a significantly increased risk of oral and pharyngeal cancer among shoe makers/cobblers, based only on two cases [21]. The results of case-control studies are also inconsistent. One study [23] observed non-significantly increased risks for both oral cavity and pharyngeal squamous cell carcinomas associated with exposure to leather dust, while another [25] found no association with hypopharynx cancers. A pooled analysis of four European studies found a non-significantly increased risk of squamous cell carcinomas of the hypopharynx and larynx group together among shoe finishers, with no association with duration of employment [19]. Concerning laryngeal cancer alone, cohort studies did not find excess risks in shoe-manufacturing workers [31–33, 35]. Two case-control studies [16, 23] observed an increased risk of laryngeal carcinoma associated with exposure to leather dust, while one study [25] did not find any association. An American case-control study observed significant risks for head and neck overall, and pharyngeal squamous cell carcinomas increasing with each decade of exposure to leather dust. Although not significant, point estimates were also elevated for cancers of the oral cavity and larynx for each successive decade of exposure [18]. In our study, non-significantly elevated risks for squamous cell carcinomas of hypopharynx, oral cavity/pharynx not specified, and larynx were associated with exposure to leather dust, but only among subjects with lower or shorter exposures; no associations were observed for cancers of the oral cavity or pharynx. Nevertheless, the average duration of exposure among cases reported in the study of Langevin et al. [18] was well above that observed in the present study (24 years vs. 5.9 years).

Two Finnish studies [21, 24] analyzed the risk for cancers of the oral cavity/pharynx, and larynx associated with exposure to leather dust estimated by the matrix FINJEM. The CEI was categorized in four categories: none, low, medium, and high exposure. No statistically significant associations were observed, although ORs were higher for the medium category (5–19 mg/m^3^-years) in the first study and for the low category (< 5 mg/m^3^-years) in the second one. Similarly, we found elevated risk of cancer of the larynx or oral cavity/pharynx (unspecified) associated with a CEI < 6 mg/m^3^-years, but not for higher exposure. This, in addition to the lack of a dose-response pattern, indicates that our study does not provide sufficient evidence for an association between HNC and exposure to leather dust.

The reliability of the present study may be considered as good. Indeed, this work provided results from one of the largest multicenter population-based case-control studies ever conducted on occupational risk factors for HNC. The large sample size allowed analyses by specific cancer sites. The collaboration with the French network of cancer registries allowed for recruitment of almost all cases with HNC in the covered geographical areas. The randomly selected control group showed a distribution of socioeconomic characteristics, and lifelong occupational exposure to leather dust comparable to that of the general population (2.2% in study controls vs. 2.4% in the French population [30]). Participation rates were satisfactory for the design of the study (80.6% for controls, and 82.5% for cases). Selection bias was therefore limited.

The specific questionnaire on leather work represented a strength of our study. The analysis was an opportunity to explore tasks such as cutting, scouring, roughing, grinding, trimming, and buffing, which are likely to expose the subject to greatest volume of leather dust. Handling of leather in boot and shoe manufacture may also entail exposure to chemicals used in the tanning and finishing processes, and other chemicals such as dyes, adhesives and solvents, for which there is evidence of carcinogenicity in humans [15]. A Russian mortality study on shoe-manufacturing workers found high leather dust concentrations (6.5–12 mg/m^3^) in the following production departments: cutting, fitting, lasting and making, and finishing [36]. In this study, leather dust was present along with solvents and chloroprene. In a Finnish study on shoe repairers, the time-weighted average concentrations of dust ranged between 0.07 and 1.0 mg/m^3^ near the roughing, scouring, and finishing machines; dust samples contained leather, polymers, finishing materials, and chromium [37]. In 1993, an American study on shoe workers [38], updated in 2006 [34], did not find any association between the cancers of oral cavity and pharynx and exposure to solvents, mostly toluene. Also, neither leather dust nor solvent exposure were associated with the risk of laryngeal cancer in a pooled analysis of two updated shoe-manufacturing cohorts [35]. In our study, non-significantly elevated risks were observed for some materials, tasks, and glues. Nevertheless, detailed analyses were difficult to perform as few workers described precisely the duration of their daily tasks and co-exposures. The small sample size in some categories reduced the statistical power required for robust associations.

Our study has some limitations. Recall bias may not be excluded, especially among those potentially underreporting their occupational history due to fatigue. Cases reported an average number of jobs slightly lower than that of the controls (4.1 vs. 4.4 [complete questionnaire]; 2.7 vs. 3.0 [shorter version]). In addition, cases more frequently answered shorter questionnaires than the controls (10.6% vs. 2.1%). Consequently, information about tasks performed and materials handled were less frequently reported by the cases due to the fact that they did not fill out the specific questionnaire on leather work. Although occupations and industries were self-reported, it is unlikely that differential reporting bias occurred because occupational exposure to leather dust is not a widely known risk factor for HNC.

Data were collected by trained interviewers using standardized questionnaires, which limited eventual differential misclassification bias. Coding occupation and industry is difficult, and often not reproducible. Therefore, coders were well-trained and blinded to case-control status. If coding errors occurred, they were probably not differential between cases and controls.

Residual confounding may not be excluded. Special attention was paid to adjustment for tobacco and alcohol consumption, and SES. Additional adjustments for body mass index, education level, exposure to asbestos and family history of HNC in first-degree relatives were made, but they did not substantially change the results. In addition, data on HPV infection and diet were not available, but it is unlikely that they would explain our results.

The exposure assessment was not based on direct measurement collected on an individual level, but was estimated through a JEM, based on job-specific averages, which does not take into account the heterogeneity of tasks within the same job title. Conversely, the JEM assigns exposure in a reproducible and automatic way, regardless of the subject status (case or control). Consequently, a non-differential misclassification bias may have occurred, which results generally in a biased estimation of the OR towards the null for dichotomous exposures [39]. However, in certain non-differential misclassification conditions with polychotomous exposure variables, estimates of ORs for categories at intermediate level can be biased away from the null [39], which could partly explain the elevated risks observed only in lower categories of exposure in the present study.

In conclusion, results from this large French population-based case-control study did not provide enough evidence in favor of a role of occupational exposure to leather dust in HNC. Further larger studies are necessary to understanding of the risks of performing specific tasks while being exposed to other agents in the leather industry.

## Additional file

Additional file 1: **Table S1.** Formula and weight for calculating the cumulative exposure index (CEI) to leather dust. **Table S2.** Odds ratios for head and neck cancer associated with occupational exposure to leather dust, stratified by sex. **Table S3.** Odds ratios for head and neck cancer associated with occupational exposure to leather dust, stratified by tobacco smoking status. **Table S4.** Odds ratios for head and neck cancer associated with occupational exposure to leather dust, stratified by alcohol drinking status. (DOCX 60 kb)

## Abbreviations

CEI: Cumulative exposure index
HNC: Head and neck cancers
HPV: Human papilloma virus
IARC: International Agency for Research on Cancer
ICD-O-3: International Classification of Diseases for Oncology, 3rd edition
ISCO: International Standard Classification of Occupations
JEM: Job exposure matrix
NAF: French Nomenclature of Activities
SES: Socioeconomic status
