# Supplementary material for: Head and neck cancer and occupational exposure to leather dust: results from the ICARE study, a French case-control study
Source: Environ Health. 2019 Mar 29;18:27. doi: 10.1186/s12940-019-0469-3 (PMC6440008; doi:10.1186/s12940-019-0469-3)
Supplement: Supplementary file 1 — Table S1. Formula and weight for calculating the cumulative exposure index (CEI) to leather dust. Table S2. Odds ratios for head and neck cancer associated with occupational exposure to leather dust, stratified by sex. Table S3. Odds ratios for head and neck cancer associated with occupational exposure to leather dust, stratified by tobacco smoking status. Table S4. Odds ratios for head and neck cancer associated with occupational exposure to leather dust, stratified by alcohol drinking status. (DOCX 60 kb) [file 12940_2019_469_MOESM1_ESM.docx]

**Supplementary Material**

**Additional file 1:**

**Article:** Head and neck cancer and occupational exposure to leather dust: results from the ICARE study, a French case-control study

**Authors:** Loredana Radoï, Fatoumata Sylla, Mireille Matrat, Christine Barul, Gwenn Menvielle, Patricia Delafosse, Isabelle Stücker, Danièle Luce, and ICARE study group.

Table S1: Formula and weight for calculating the cumulative exposure index (CEI) to leather dust.

Table S2: Odds ratios for head and neck cancer associated with occupational exposure to leather dust, stratified by sex.

Table S3: Odds ratios for head and neck cancer associated with occupational exposure to leather dust, stratified by tobacco smoking status.

Table S4: Odds ratios for head and neck cancer associated with occupational exposure to leather dust, stratified by alcohol drinking status.

**Table S1.** Formula and weight for calculating the cumulative exposure index (CEI) to leather dust.

CEI = Σ lifetime (weighted duration * weighted probability * weighted level)

| **Leather dust** | | |
| --- | --- | --- |
| **Parameter** | **Definition** | **Weight** |
| **Probability of exposure** | **% of workers of the employment concerned** |  |
| 0 | P≤ 1% | 0 |
| 1 | 1<P≤ 10% | 0.05 |
| 2 | 10<P≤ 50% | 0.3 |
| 3 | 50<P≤ 90% | 0.7 |
| 4 | P>90% | 0.95 |
| **Level of exposure** | **Concentration in mg/m^3^** |  |
| 0 | N = 0 | 0 |
| 1 | N< 1 | 0.5 |
| 2 | 1≤N< 3 | 2 |
| 3 | 3≤N<7 | 5 |
| 4 | N≥7 | 10 |

**Table S2. Odds ratios for head and neck cancer associated with occupational exposure to leather dust, stratified by sex.**

|  |  | ***All subjects (n=5716)*** | | |  |  | ***Males (n=4642)*** | | ***Females (n=1074)*** | | | |
| --- | --- | --- | --- | --- | --- | --- | --- | --- | --- | --- | --- | --- |
|  |  | **Cases\|Controls** | | **OR^1^  [95% CI]** |  | **Cases\|Controls** | | **OR^1^ [95% CI]** | |  | **Cases\|Controls** | **OR^1^ [95% CI]** |
| **Exposure to leather dust** | |  |  |  |  |  | |  | |  |  |  |
| Never exposed ^*^ | |  | 2087\|3476 | reference |  | 1797\|2728 | | reference | |  | 290\|748 | reference |
| Ever exposed | |  | 57\|75 | 0.99 [0.62-1.56] |  | 50\|50 | | 1.08 [0.65-1.80] | |  | 7\|25 | 0.65 [0.21-2.01] |
| **Maximum probability of exposure (%)** | | | |  |  |  | |  | |  |  |  |
| ≤ 50 | |  | 26\|32 | 1.17 [0.59-2.31] |  | 23\|24 | | 1.31 [0.63-2.70] | |  | 3\|8 | 0.47 [0.05-4.32] |
| > 50 | |  | 31\|43 | 0.86 [0.47-1.58] |  | 27\|26 | | 0.90 [0.44-1.82] | |  | 4\|17 | 0.72 [0.20-2.57] |
| **Maximal level of exposure (mg/m^3^)** | | | |  |  |  | |  | |  |  |  |
| < 3 | |  | 41\|50 | 1.09 [0.63-1.89] |  | 37\|30 | | 1.41 [0.75-2.65] | |  | 4\|20 | 0.37 [0.08-1.74] |
| ≥ 3 | |  | 16\|25 | 0.79 [0.35-1.77] |  | 13\|20 | | 0.63 [0.26-1.53] | |  | 3\|5 | 1.52 [0.27-8.53] |
| **Cumulative duration of exposure (years)** | | | |  |  |  | |  | |  |  |  |
| ≤ 7 | |  | 45\|43 | 1.22 [0.70-2.14] |  | 39\|29 | | 1.39 [0.74-2.63] | |  | 6\|14 | 0.81 [0.21-3.06] |
| > 7 | |  | 12\|32 | 0.63 [0.28-1.44] |  | 11\|21 | | 0.65 [0.26-1.60] | |  | 1\|11 | 0.39 [0.04-3.50] |
| **Cumulative exposure index (mg/m^3^-years)** | | | |  |  |  | |  | |  |  |  |
| ≤ 6 | |  | 37\|38 | 1.43 [0.78-2.61] |  | 32\|25 | | 1.57 [0.80-3.08] | |  | 5\|13 | 0.93 [0.20-4.32] |
| > 6 | |  | 20\|37 | 0.60 [0.29-1.22] |  | 18\|25 | | 0.63 [0.28-1.40] | |  | 2\|12 | 0.44 [0.08-2.36] |

^*^ Never exposed = reference category for all variables presented in this table.

^1^ ORs (odds ratios) adjusted for age, sex, area of residence, socioeconomic status, and tobacco and alcohol consumption.

**Table S3. Odds ratios for head and neck cancer associated with occupational exposure to leather dust, stratified by tobacco smoking status.**

|  | ***All subjects (n=5716)*** | |  |  | ***Never smokers (n=1372) Ever smokers (n=4322)*** | | | | |
| --- | --- | --- | --- | --- | --- | --- | --- | --- | --- |
|  | **Cases\|Controls** | **OR^1^ [95% CI]** |  | **Cases\|Controls** | | **OR^1^ [95% CI]** |  | **Cases\|Controls** | **OR ^1^ [95% CI]** |
| **Exposure to leather dust** |  |  |  |  | |  |  |  |  |
| Never exposed ^*^ | 2087\|3476 | reference |  | 107\|1232 | | reference |  | 1973\|2232 | reference |
| Ever exposed | 57\|75 | 0.99 [0.62-1.56] |  | 2\|28 | | 0.69 [0.15-3.12] |  | 55\|47 | 1.11 [0.68-1.80] |
| **Maximum probability of exposure (%)** | |  |  |  | |  |  |  |  |
| ≤ 50 | 26\|32 | 1.17 [0.59-2.31] |  | 0\|10 | | - |  | 26\|22 | 1.50 [0.73-3.07] |
| > 50 | 31\|43 | 0.86 [0.47-1.58] |  | 2\|18 | | 1.13 [0.24-5.25] |  | 29\|25 | 0.87 [0.46-1.66] |
| **Maximal level of exposure (mg/m^3^)** | |  |  |  | |  |  |  |  |
| < 3 | 41\|50 | 1.09 [0.63-1.89] |  | 1\|19 | | 0.51 [0.06-4.05] |  | 40\|31 | 1.39 [0.76-2.51] |
| ≥ 3 | 16\|25 | 0.79 [0.35-1.77] |  | 1\|9 | | 1.10 [0.12-9.72] |  | 15\|16 | 0.72 [0.31-1.64] |
| **Cumulative duration of exposure (years)** | |  |  |  | |  |  |  |  |
| ≤ 7 | 45\|43 | 1.22 [0.70-2.14] |  | 1\|16 | | 0.85 [0.10-6.83] |  | 44\|27 | 1.41 [0.78-2.57] |
| > 7 | 12\|32 | 0.63 [0.28-1.44] |  | 1\|12 | | 0.58 [0.07-4.76] |  | 11\|20 | 0.66 [0.27-1.60] |
| **Cumulative exposure index (mg/m^3^-years)** | |  |  |  | |  |  |  |  |
| ≤ 6 | 37\|38 | 1.43 [0.78-2.61] |  | 1\|14 | | 0.69 [0.08-5.73] |  | 36\|24 | 1.73 [0.90-3.32] |
| > 6 | 20\|37 | 0.60 [0.29-1.22] |  | 1\|14 | | 0.69 [0.08-5.72] |  | 19\|23 | 0.63 [0.30-1.31] |

^*^ Never exposed = reference category for all variables presented in this table.

^1^ ORs (odds ratios) adjusted for age, sex, area of residence, socioeconomic status, and tobacco and alcohol consumption.

**Table S4. Odds ratios for head and neck cancer associated with occupational exposure to leather dust, stratified by alcohol drinking status.**

|  | ***All subjects (n=5716)*** | | | |  | |  | | ***Never drinkers (n=409) Drinkers (n=5231)*** | | | | | | | |
| --- | --- | --- | --- | --- | --- | --- | --- | --- | --- | --- | --- | --- | --- | --- | --- | --- |
|  | **Cases\|Controls** | | **OR^1^ [95% CI]** | |  | | **Cases\|Controls** | | | **OR^1^ [95% CI]** | |  | **Cases\|Controls** | | **OR ^1^ [95% CI]** | |
| **Exposure to leather dust** | |  | |  | |  | |  | | |  |  | |  | |  |
| Never exposed ^*^ | 2087\|3476 | | reference | |  | | 98\|299 | | | reference | |  | 1943\|3157 | | reference | |
| Ever exposed | 57\|75 | | 0.99 [0.62-1.56] | |  | | 3\|6 | | | 1.67 [0.28-9.65] | |  | 54\|68 | | 1.06 [0.66-1.70] | |
| **Maximum probability of exposure (%)** | | |  | |  | |  | | |  | |  |  | |  | |
| ≤ 50 | 26\|32 | | 1.17 [0.59-2.31] | |  | | 1\|1 | | | 10.55 [0.30-366.65] | |  | 25\|30 | | 1.21 [0.61-2.41] | |
| > 50 | 31\|43 | | 0.86 [0.47-1.58] | |  | | 2\|5 | | | 0.93 [0.11-7.36] | |  | 29\|38 | | 0.95 [0.50-1.79] | |
| **Maximal level of exposure (mg/m^3^)** |  | |  | |  | |  | | |  | |  |  | |  |  |
| < 3 | 41\|50 | | 1.09 [0.63-1.89] | |  | | 1\|3 | | | 3.51 [0.22-54.44] | |  | 40\|46 | | 1.28 [0.73-2.25] | |
| ≥ 3 | 16\|25 | | 0.79 [0.35-1.77] | |  | | 2\|3 | | | 1.08 [0.12-9.69] | |  | 14\|22 | | 0.69 [0.30-1.62] | |
| **Cumulative duration of exposure (years)** | | |  | |  | |  | | |  | |  |  | |  | |
| ≤ 7 | 45\|43 | | 1.22 [0.70-2.14] | |  | | 2\|3 | | | 1.24 [0.13-11.37] | |  | 43\|39 | | 1.38 [0.77-2.49] | |
| > 7 | 12\|32 | | 0.63 [0.28-1.44] | |  | | 1\|3 | | | 2.64 [0.19-36.74] | |  | 11\|29 | | 0.63 [0.27-1.46] | |
| **Cumulative exposure index (mg/m^3^-years)** | | |  | |  | |  | | |  | |  |  | |  |  |
| ≤ 6 | 37\|38 | | 1.43 [0.78-2.61] | |  | | 1\|1 | | | 10.55 [0.30-366.65] | |  | 36\|36 | | 1.48 [0.80-2.71] | |
| > 6 | 20\|37 | | 0.60 [0.29-1.22] | |  | | 2\|5 | | | 0.93 [0.11-7.36] | |  | 18\|32 | | 0.65 [0.30-1.37] | |

^*^ Never exposed = reference category for all variables presented in this table.

^1^ ORs (odds ratios) adjusted for age, sex, area of residence, socioeconomic status, and tobacco and alcohol consumption.
